# Neutral Electrolyzed Water as Sanitizer Solution in Fresh Foods: The Strawberry as a Study Model

**DOI:** 10.3390/foods15050800

**Published:** 2026-02-24

**Authors:** Juan Carlos Ramírez-Orejel, Patricia Ventura-Torres, Karen Arisbeth Alvarez-Cruz, Ketzalzin Vazquez-Hernández, Patricia Severiano-Pérez, José Alberto Cano-Buendía

**Affiliations:** 1 Department of Animal Nutrition and Biochemistry, Facultad de Medicina Veterinaria y Zootecnia, Universidad Nacional Autónoma de México (UNAM), Ciudad Universitaria, Mexico City 04510, Mexico; jrorejel@unam.mx (J.C.R.-O.); arisbeth.alvarez.x@gmail.com (K.A.A.-C.); ketzvh@gmail.com (K.V.-H.); 2 Department of Micriobiology and Immunology, Facultad de Medicina Veterinaria y Zootecnia, Universidad Nacional Autónoma de México (UNAM), Ciudad Universitaria, Mexico City 04510, Mexico; patyvenntura@gmail.com; 3 Department of Food and Biotechnology, Facultad de Química, Universidad Nacional Autónoma de México (UNAM), Ciudad Universitaria, Mexico City 04510, Mexico; pspmex1@unam.mx

**Keywords:** electrolyzed water, strawberries, disinfection, shelf-life

## Abstract

Strawberries are a very fresh product, but they pose food safety concerns. In the present work, the conservative effect of Neutral Electrolyzed Water (NEW) on strawberries was evaluated on contaminated berries with *Salmonella* Typhimurium ATCC 13311 and *Escherichia coli* O157:H7. In addition, the treated fruit underwent physicochemical analysis, evaluating color, pH, titrable acidity, reducing sugar, soluble solids, total phenolic compounds, and vitamin C content. The effects of NEW on strawberries were compared with those caused after treatment with NaClO and saline solutions; all evaluated solutions were applied by spraying. Subsequently, sensory analysis was performed to identify differences between treatments. NEW and NaClO showed similar in vitro bactericidal effects against *E. coli*, and for *Salmonella* Typhimurium, NEW and NaClO decreased their counts to 9.3 log CFU/mL and 5.16 log CFU/mL, respectively. When both solutions were sprayed on contaminated strawberries, *S.* Typhimurium counts were decreased to 1.9 log CFU/mL and 1.29 log CFU/mL with NEW and NaClO, respectively, and the *E. coli* counts were 2.12 log CFU/mL and 1.31 log CFU/mL, respectively. This work includes physicochemical properties of treated strawberries and a sensory study to identify how treatments affect fruit characteristics. The physicochemical properties of the treated strawberries were not affected statistically significantly. Sensory analysis revealed that although oxidizing sanitizers (NEW or NaClO) modified the overall sensory profile of strawberries, NEW-treated samples preserved key desirable attributes such as uniform appearance, fresh odor intensity, and characteristic strawberry flavor, without generating pronounced off-odors or unacceptable taste alterations. These results indicate that NEW is a promising postharvest sanitizing alternative capable of ensuring microbial safety while maintaining sensory quality relevant to consumer acceptance.

## 1. Introduction

Strawberry (*Fragaria x ananassa*) is one of the most consumed berries worldwide. For centuries, it has been part of the human diet thanks to its attractive color, appearance, and flavor [1]; in addition, it constitutes one of the main dietary sources of bioactive compounds such as vitamin C, folic acid, lutein, zeaxanthin, choline and phenolic compounds (phenolic acids, flavonoids, ellagitannins and anthocyanins), organic acids, and dietary fiber and minerals (calcium, iron, magnesium, phosphorus, potassium, sodium, zinc, copper, magnesium, and selenium) [2]. These compounds offer multiple health benefits; for example, they reduce blood pressure and prevent cardiovascular diseases, strengthen the immune system, prevent and reduce inflammation, and reduce oxidative stress, and they have been reported to reduce the risk of various types of cancer and chronic degenerative diseases such as diabetes and obesity [3,4,5].

Strawberries are highly perishable fruits that are commonly consumed fresh or minimally processed. Their rough surface morphology, high respiration rate, and close contact with soil during cultivation favor the attachment and survival of pathogenic microorganisms, increasing the risk of foodborne illness [6,7]. This risk is particularly relevant for organically produced strawberries, where the use of animal manure as fertilizer may result in higher microbial loads compared to conventional production systems [8]. Several outbreaks associated with *Escherichia coli* O157:H7 and *Salmonella* spp. linked to the consumption of fresh berries have been documented, highlighting the need for effective postharvest sanitization strategies that ensure microbial safety without compromising fruit quality [9,10]. In recent years, one well-known and serious outbreak of *Escherichia coli* O157:H7 has been documented related to the consumption of contaminated strawberries in Oregon in 2011; it involved 15 human cases and 2 human deaths. Another case of Salmonella infection was surveilled by the Food and Drug Administration (FDA), reporting that 1 in 143 lots of imported strawberries into the U.S. was contaminated by Salmonella [7]. Although most foodborne outbreaks came from conventional strawberries, the use of animal manure as a natural fertilizer might result in even higher contamination levels of microorganisms in strawberries from organic systems than those from conventional farms, making the sanitation process even more important to ensure the safety of organic strawberries.

Conventional sanitizing agents such as sodium hypochlorite, chlorine dioxide, and ozone are widely used to reduce microbial contamination in fresh produce. However, their application presents several limitations, including the formation of potentially harmful disinfection by-products, residual chlorine on food surfaces, and negative effects on sensory and nutritional quality [6]. In addition, increasing regulatory restrictions and consumer concerns regarding chemical residues have driven the search for alternative, environmentally friendly, and residue-free sanitizing technologies suitable for fresh and ready-to-eat fruits such as strawberries [7,11].

Electrolyzed water (EW) has emerged as a promising alternative for food disinfection due to its strong antimicrobial activity, low cost, ease of on-site generation, and minimal chemical residues [7,12]. Depending on electrolysis conditions, EW can be classified as acidic, slightly acidic, neutral, or alkaline, each exhibiting different physicochemical properties and antimicrobial mechanisms [13]. Although acidic and slightly acidic EW have demonstrated high bactericidal efficacy, their low pH has been associated with potential adverse effects on the physicochemical stability and sensory quality of delicate fruits such as strawberries [13,14]. The application of EW in food preservation focuses primarily on the microbial control of poultry, meat, and seafood [7,15]. However, it has also shown effective results in fresh fruits and ready-to-eat vegetables like lettuce [16,17], pitaya fruit [18], rambutan fruit [19], and Hass avocado [20]; studies have proven that EW helps decrease the spoiled bacterial load [21], preserve the postharvest quality, and improve the storage times of different foods.

Neutral electrolyzed water (NEW), characterized by a near-neutral pH and high oxidation–reduction potential, has gained attention as a sanitizing alternative that balances antimicrobial efficacy with quality preservation [7,22]. Unlike acidic or alkaline EW, NEW reduces the risk of pH-induced tissue damage while maintaining sufficient oxidative capacity to inactivate foodborne pathogens on fresh produce surfaces [13,22]. Several studies have reported that NEW effectively reduces microbial contamination on fruits and vegetables while exerting limited effects on physicochemical and sensory attributes, making it particularly suitable for application on fresh and ready-to-eat fruits [23].

Therefore, the aim of the present study was to evaluate the effectiveness of neutral electrolyzed water as a sanitizing treatment for fresh strawberries artificially contaminated with *Salmonella* Typhimurium and *Escherichia coli* O157:H7. In addition to microbial reduction, this work assessed the impact of NEW on physicochemical properties (pH, titratable acidity, soluble solids, total phenolic compounds, vitamin C content and color) and sensory attributes, recognizing that consumer acceptance is a critical factor in the implementation of postharvest sanitizing technologies. Despite the existence of many studies of the impact of EW and bacterial reduction, to the best of our knowledge, this is the first work that includes a physicochemical and sensory analysis of treated strawberries with neutral electrolyzed water.

## 2. Materials and Methods

### 2.1. Fruit Acquisition

Strawberries (*Fragaria x ananassa* cultivar Driscoll) were purchased at a local market in the south of Mexico City; the strawberries were packed into closed clamshells. They were inspected for spoiling, and unwanted ones were discarded. They were randomly mixed in groups of 50 and stored in sterile plastic bags at 4 °C until use.

### 2.2. Reagents

Neutral electrolyzed water (NEW) was supplied by a commercial manufacturer located in Mexico (Esteripharma S.A. de C.V.), selected based on its availability for local food producers and its compliance with national regulations for use in different foods. The use of a locally produced NEW was intended to enhance the practical relevance and potential scalability of the sanitizing treatment under conditions representative of the fresh produce industry, where postharvest disinfection technologies must be accessible, cost-effective, and suitable for on-site use.

The NEW used in this study was generated through the electrolysis of a sodium chloride solution in a chamber without membrane and characterized by a near-neutral pH and high oxidation–reduction potential (ORP), as specified by the supplier. These characteristics are consistent with those reported for NEW systems approved for food processing applications, which combine antimicrobial efficacy with reduced corrosiveness and lower risk of quality degradation compared to acidic or alkaline electrolyzed water.

The physicochemical properties of NEW, including pH and ORP, were verified prior to its application. The concentration of sodium hypochlorite (Quimica Rique, Cat. No. 7681-52-9, Ecatepec de Morelos, Mexico) was adjusted to 100 ppm because chlorine is used in concentrations of 50 to 150 ppm in the fruit and vegetable industry, and sodium hypochlorite was considered a disinfectant control. Saline solution (SS) (0.9% NaCl) (Química Suastes S.A. de C.V., Cat. No. 6845, Mexico City, Mexico) was considered a washing control because NEW is made with NaCl and tap water was not used because it has chlorine. All solutions were diluted/adjusted with sterile distilled water. pH and ORP values were measured using a portable pH/ORP/temperature combo tester (Cat. No. HI98121, Hanna Instruments, Woonsocket, RI, USA) following the manufacturer’s instructions. The chlorine concentration was measured using a portable chlorine photometer (Cat. No. HI96771, Hanna Instruments, Woonsocket, RI, USA), and the iodometric method [24] was used to evaluate free chlorine content.

### 2.3. Bacterial Strain and Inoculum Preparation

*Escherichia coli* O157:H7 and *Salmonella* Typhimurium were obtained from the American Type Culture Collection (ATCC 43888 and ATCC 13311). Bacteria were grown on Trypticase Soy Agar (TSA) (Cat. No. 210800, BD Bioxon, México City, México) at 37 °C for 24 h. Individual colonies of *E. coli* O157:H7 and *Salmonella* Typhimurium were subsequently grown in 50 mL of trypticase soy broth (TSB) (Cat. No. 7381, MCD LAB, Tultitlán de Mariano Escobedo, México) at 37 °C for 16 h at 200 rpm. Viable cell numbers were determined using the aerobic plate count, where 10-fold serial dilutions were performed in PBS for a final volume of 10 mL. One hundred microliters of each dilution was plated onto a Petri dish containing 15 mL of TSA. The plates were incubated overnight at 37 °C, and colony-forming units were counted, adjusting for the dilution factor [25,26]. The number of bacteria was expressed in CFU/mL.

### 2.4. Bactericidal In Vitro Test

Mexican Standard NMX-BB-040-SCFI-1999 was performed [27]. Briefly, after evaluating all working solutions (NEW, NaClO and SS), 99 mL of each solution was transferred to a sterile 250 mL Erlenmeyer flask. The solutions were shaken, and 1 mL of *E. coli* O157:H7 or *Salmonella* Typhimurium inoculum (~1 × 10^9^ CFU/mL) was added to facilitate their incorporation. After 30 s, 1 mL of the mixture was transferred to a tube with 9 mL of 0.1% peptone water (used as neutralizing solution) and subsequently mixed. Serial decimal dilutions were prepared, and 0.1 mL of each dilution was seeded on Petri dishes with TSA. The plates were incubated for 48 h at 37 °C. The CFUs were subsequently determined. Inoculum treatment and serial dilutions were performed at room temperature. Experiments were performed in triplicate.

Bacterial percentage reduction was calculated using Equation (1).

(1) $$R\left(\%\right)=100-\left(\frac{\left(A\times 100\right)}{B}\right)$$

where

A represents viable bacteria after treatment;

B represents viable bacteria with no treatment.

### 2.5. Bactericidal Test in Strawberries

Inoculum was prepared using an overnight culture of *E. coli* O157:H7 or *Salmonella* Typhimurium. The contamination inoculum was prepared using 0.1% peptone water to adjust the bacteria to 10^7^ CFU/mL. Subsequently, 300 strawberries were divided into two groups: The first group of 150 berries was immersed in the *E. coli* O157:H7 inoculum for 15 min; then, the contaminated fruits were held in a sterile plastic colander for 5 min to allow the inoculum to drain into the container. This was performed in a biosafety cabinet at room temperature. The second group of 150 fruits was submerged in the *Salmonella* Typhimurium inoculum and underwent the same processing as the first group.

#### 2.5.1. Strawberry Treatment

Artificially contaminated strawberries were divided into three groups containing 50 pieces each. The groups were labeled as NEW, NaClO, and SS. Spray treatment was applied because it uses less water than dipping treatment. Working solutions were applied using spray bottles containing 25 mL of each solution to each group. The strawberries were turned over when half of the treatment was applied, and the remaining solution was used, meaning 1 mL of treatment solution per strawberry. The working solutions were in contact with the strawberry samples for 1 min because the Mexican normativity [27] evaluates bactericidal effect in 30 s and fruit transportation uses conveyor belts that help move strawberries fast.

After, strawberries were individually deposited into a plastic bag containing 50 mL of 0.1% peptone water as an inactivation solution, allowing the collection of surviving bacteria after treatment. The strawberries were manually rubbed for 1 min, and 1 mL aliquot was taken from the plastic bags and used for plate counting. The strawberries were individually placed into new plastic bags and kept at 4 °C. The number of viable cells was determined on days 1 and 8 using the aerobic plate count methodology. Because it has been reported that spoilage microbiota contributes to the fresh produce shelf life, we decided to count all present bacteria on the strawberries.

#### 2.5.2. Non-Contaminated Study

To determine the amount of natural bacterial contamination of strawberries, a group of 50 strawberries was treated as described in the Bacterial recovery after treatment section. The total viable aerobic counts were determined using TSA plates, and the presence of *Salmonella* spp. (white colonies) or *E. coli* (pink colonies) was determined using plates of MacConkey agar (Cat. No. 210900, BD Bioxon, México City, México).

### 2.6. Physicochemical Analysis

A new batch of uncontaminated strawberries (150 berries) was treated with evaluated solutions as described in the Strawberry treatment section and kept in plastic bags at 4 °C for 5 days. It was decided to evaluate on the first 5 days after treatment because EW, as an oxidizing solution, has a high ORP which could affect some characteristics fast and a similar study that used acid and alkaline electrolyzed water monitored their impact for 7 days [28]. Their physicochemical properties [pH, total acidity, soluble solids °Bx, total phenolic content, vitamin C content, color (*L*, *a* and *b*) and sensorial analysis] were determined as described below.

#### 2.6.1. pH

pH was determined according to AOAC method 981.12 [29] using a pH/ORP meter (Cat. No HI 2211, Hanna Instruments, Woonsocket, RI, USA). Strawberry pulp (10 g) was weighed for each treatment with disinfectant solution, and distilled water (30 mL) was added. The mixture was homogenized using a blender (Intertek, WF2211214, London, UK), transferred into a beaker, and then kept under constant stirring. Finally, a pH meter electrode was immersed in the solution until completely covered.

#### 2.6.2. Titratable Acidity

Titratable acidity was determined according to the AOAC method 942.15. Citric acid content was expressed using the citric acid monohydrate 0.070 factor. Briefly, 10 g of homogenized strawberries (obtained using the same methodology described in the pH section) was diluted in 100 mL of boiled water. Subsequently, a titration was performed with 0.1 N NaOH, using 0.3 mL of phenolphthalein until a pink color persisted for more than 30 s. The results were given as % of citric acid per 100 g of fruit, as citric acid is the primary organic acid present in strawberries.

#### 2.6.3. Reducing Sugars

From homogenized berries, 1 mL of the aqueous sample solution was taken, and 1 mL of DNS reagent was added. The mixture was heated for 5 min in a boiling water bath and then cooled and diluted with 10 mL of distilled water. Absorbance was read at 540 nm using a UV/VIS spectrometer (Perkin Elmer, Lambda 2S, Waltham, MA, USA); a blank was considered the reagent mixture and water was treated as the sample. The reducing sugars were calculated using a glucose standard curve and were given as % of reducing sugars per 100 g of fruit.

#### 2.6.4. Soluble Solids

AOAC method 932.12 was used. Total soluble solids, expressed as °Brix, were recorded using a Wincom VBR32 portable refractometer on strawberry pulp from each treatment.

#### 2.6.5. Total Phenolic Compounds

Strawberry samples (10 g) were extracted three times in a solvent mixture (100 mL the first time, 50 mL the next two times) comprising methanol/water/acetic acid (70:30:5). The homogenate was filtered using filter paper (Whatman No 1). Analysis was performed according to [3]; 0.25 mL of the extract was mixed with 0.25 mL Folin–Ciocalteu reagent, and 2 mL of distilled water was added and incubated for 3 min at room temperature. Then, 0.25 mL of a saturated sodium carbonate (Cat. No. 3506-1, J.T. Baker, Estado deMéxico, México) solution was added, and the mixture was incubated at 37 °C in a water bath for 30 min. The absorbance was measured at 750 nm. The results are expressed as mg of gallic acid/100 g of sample.

#### 2.6.6. Vitamin C Content

Because the vitamin C content in strawberries varies with harvest time, season, location, and growing conditions [30,31,32,33], it is important to quantify it and detect whether EW affects its presence in strawberries. The protocol followed was the AOAC method 967.21. Fresh strawberry juice was prepared and then filtered. Then, 100 mL of the juice obtained was mixed with an equal volume of HPO3—AcHO solution. Samples were titrated in triplicate with indophenol [34]. Indophenol is accepted by AOAC for vitamin C determination.

#### 2.6.7. Color

An instrumental evaluation of strawberry color was performed using a pre-calibrated portable colorimeter (Konica Minolta CM-600d, Ramsey, NJ, USA). Five random zones were used to measure the lab color space (CIELab*). The ΔE value of each treatment was calculated [35].

### 2.7. Sensorial Analysis

The strawberries were divided into groups of 25 pieces each, labeled as NEW, NaClO, and colloidal silver. Strawberries were treated with the evaluated solutions as described above. Colloidal silver was included in the sensory evaluation to provide a reference point for sensory changes associated with alternative sanitizing agents, without implying a direct comparison with the main disinfectants evaluated in this study [36,37]. Colloidal silver is a safe antimicrobial used in strawberries [38], and it is used in household and small-scale food sanitation practices in Mexico and other countries. There is no reporting on the impact of colloidal silver in sensory analysis.

The saline control group was not included in the sensory evaluation because saline solutions may directly affect taste by introducing a salty perception that is not representative of sanitizing treatments [39], and because undisinfected strawberries cannot be authorized for a sensory evaluation by the Ethical Committee. In contrast, NEW and NaClO were included because they are commonly used disinfectants in fresh produce processing, and evaluating their potential impact on sensory attributes is relevant for practical application and consumer acceptance.

The ionized silver disinfectant solution was prepared following the manufacturer’s recommendations, with a final silver concentration of 1.17 ppm in distilled water. It is important to highlight that the use of disinfectants should not compromise the sensory characteristics (appearance, aroma, taste, or texture) of foods.

The sensory profile was generated using the modified Flash Profile, which is a rapid methodology carried out by judges previously trained in a conventional descriptive methodology for foods other than the one under study (strawberries). It allows better discrimination and consensus of results [40,41]. Food was analyzed using the configurations (attributes) used in this study and in other sensory profiles that used rapid methods [42,43,44].

The sensory attributes evaluated in the modified Flash Profile analysis included appearance, odor, taste, aftertaste, and texture, which were freely generated and agreed upon by the trained panel during the initial consensus session, following standard Flash Profile methodology.

For appearance, attributes such as uniformity, brightness, and color homogeneity were considered. Odor attributes included strawberry aroma intensity, freshness, fruity, citrus, herbaceous, and musty notes. Taste descriptors comprised sweetness, strawberry flavor intensity, fruity and herbaceous notes, as well as the presence of off-flavors. Aftertaste descriptors included persistence and metallic notes, while texture attributes included firmness, crunchiness, chewiness, graininess, astringency, and juiciness.

The evaluation was conducted by a panel of judges trained at the Sensory Evaluation Research Laboratory of the School of Chemistry, UNAM. The panel consisted of 13 judges: 11 women and 2 men, aged between 21 and 25 years. To ensure proper evaluation, eliminate potential distractions, and facilitate communication between judges, individual booths were used in accordance with ISO 8589 [45]. During the evaluations, strawberries were presented on white plates with a three-digit numerical code (randomly assigned).

Ethical approval for the involvement of human judges in this study was granted by the Comisión the Ética y Resposabilidad Científica de la Facultad de Ciencias, with approval Code PI_2021_11_18_Severiano, on 24 January 2022, under the program “Apoyo para desarrollar y aplicar pruebas de Evaluación Sensorial”.

Participants provided informed consent by stating the following: “I am aware that my responses are confidential, and I agree to participate in this survey”. They were able to withdraw from the survey at any time without giving a reason. The products tested were safe for consumption.

For statistical analysis of the modified Flash Profile data, Generalized Procrustes Analysis (GPA) was applied to obtain a consensus sensory configuration among judges. Principal Component Analysis (PCA) was subsequently performed on the consensus matrix to explain the variance of the sensory dimensions [46,47,48].

### 2.8. Statistical Analysis

Data were presented as mean ± standard deviation (SD). The obtained results were analyzed by two-factor analysis of variance (ANOVA) and Tukey’s mean difference test, using Graphpad Prism 6 software with a 95% confidence level. *p*-values < 0.05 were considered as statistically significant. Different lowercase letters in the tables indicate significant differences.

## 3. Results and Discussion

### 3.1. Characteristics of the Solutions Used

All solutions evaluated were analyzed prior to use to characterize their pH, ORP, and available chlorine concentration properties, which are related to their antimicrobial efficacy. The pH of NEW and NaClO was 6.24 ± 0.03 and 7.45 ± 0.01, respectively; SS showed a pH of 6.9 ± 0.06. NEW had the highest ORP value (878.56 ± 0.88 mV), followed by NaClO (820.11 ± 1.74 mV); SS had the lowest value (380 ± 1.43 mV), which was expected due to its lack of oxidizing elements. It has been reported that ORP modifies bacterial metabolic fluxes and ATP production, causing changes in the electron flow due to its oxidizing capacity [49]. Moreover, NEW had a free chlorine value of 54.96 ± 0.58 ppm. The main component of NEW is hypochlorous acid; however, other components (~5%) such as hypochlorite ions and traces of chlorine, are present at lower levels.

The concentrations of NaClO was adjusted to 100 ppm to be within the range considered by the FDA and FAO [10,50] for the disinfection of fruits and vegetables, between 100 and 200 ppm. NEW concentration was not adjusted. The free chlorine concentration in SS was lower than that observed in our detection method (Table 1).

### 3.2. In Vitro Bactericidal Effect

The bactericidal effects of NEW and NaClO were compared to the results obtained with the SS treatment. The evaluated solutions caused a decrease in the number of viable counts. NEW decreased *Salmonella* Typhimurium counts by more than 6.4 log CFU/mL and *E. coli* O157:H7 counts by more than 6.3 log CFU/mL compared to the SS treatment. The NaClO treatment achieved a decrease in bacterial counts of 4.2 log CFU/mL and of more than 6.3 log CFU/mL for *S.* Typhimurium and *E. coli* O157:H7, respectively. Both groups were significantly different from the SS treatment (*p* < 0.0001) (Table 2). Due to the detection limit of the microbiology test (log CFU/mL = 3), the bactericidal effect could be higher for NEW against both bacterial strains and for *E. coli* when treated with NaClO. However, this data showed that *Salmonella* Typhimurium was resistant to the effect of NaClO.

### 3.3. Natural Bacterial Counts in Strawberries

Non-treated strawberries were analyzed for the presence of *Salmonella* spp. or *Escherichia coli*. No bacterial growth was detected in MacConkey agar plates. However, the natural (original) aerobic bacterial count was 3.3 log CFU/g (2 × 10^3^ CFU/g). As natural bacteria also contribute to the spoilage of strawberries, we decided not to administer additional treatment, which would eliminate the natural bacteria, and the following analyses were performed using TSA plates to account for natural original contaminant bacteria.

### 3.4. Bacterial Reduction in Strawberries

**Figure 1:**
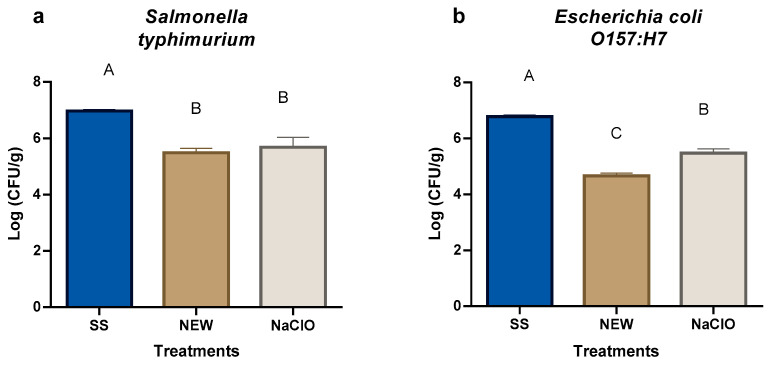
Bacterial numbers after treating strawberries contaminated with (**a**) *Salmonella* Thyphimurium and (**b**) *Escherichia coli* O157:H7. Data points reflect the mean of triplicate measurements ± standard deviation. Different letters indicate significant differences (*p* ≤ 0.05).

The use of NEW (54.96 ppm) resulted in a 1.49 log CFU/g reduction in the *Salmonella* Typhimurium count (Figure 1a), while sodium hypochlorite (NaClO, 100 ppm) treatment generated a significant reduction of 1.29 log CFU/g. These results indicate that both disinfectants were effective against *S.* Typhimurium when applied by spraying on strawberry surfaces (Figure S1).

In the case of *Escherichia coli* O157:H7, NEW achieved a bacterial reduction of 2.12 log CFU/g (Figure 1b), whereas the NaClO treatment generated a bacterial count reduction of 1.31 log CFU/g. Both numbers are significantly different from those of the SS treatment (Figure S2). The results show that *Escherichia coli* O157:H7 is more susceptible to disinfectant treatments than *S.* Typhimurium. *Escherichia coli* O157:H7 has been reported to be susceptible to chlorine treatment when strawberries were washed for 5 min [51].

NEW showed a higher bactericidal effect than NaClO, and the concentration was higher for NaClO (100 ppm) than NEW (54.96 ppm). This effect has been reported previously, claiming 80 times stronger bactericidal activity of EW [52,53] than ^−^ClO, even with the presence of organic material [54]. The bactericidal activity is related to the summatory effects of ORP, chlorine content (Cl^−^, HClO, ClO^−^, HCl) and pH [55,56,57,58]. These properties favor NEW’s bactericidal activity over NaClO.

Overall, *E. coli* O157:H7 showed greater susceptibility to the disinfectant treatments than *S.* Typhimurium. Similar trends have been reported in previous studies, where electrolyzed water treatments produced higher inactivation levels of *E. coli* compared to *Salmonella* on strawberries and other fresh fruits [14]. It has been reported that there is similar resistance of *S*. Typhimurium against NaOCl at 500 ppm [21] due to biofilm formation.

The effectiveness of electrolyzed water as a sanitizing agent for fresh produce has been widely documented. Reductions ranging from 1 to 3 log CFU/g for *Salmonella* spp. and *E. coli* have been reported on fruits and vegetables treated with neutral or slightly acidic electrolyzed water, depending on treatment conditions and exposure time [7,13]. In some studies, higher reductions than those observed in the present work were achieved, which may be attributed to longer contact times or immersion treatments rather than spraying applications.

The lower susceptibility of *S.* Typhimurium compared to *E. coli* O157:H7 may be explained by biological differences between these microorganisms. *Salmonella enterica* has a greater ability to adhere to produce surfaces and form biofilms [22], which can limit the penetration of sanitizing agents and increase resistance to oxidative disinfectants [59]. In contrast, *E. coli* cells are generally more susceptible to chlorine-based and electrolyzed water treatments due to differences in cell envelope structure, stress response mechanisms, and weaker attachment to fruit epidermal tissues. These factors, combined with the complex surface morphology of strawberries, likely contribute to the observed differences in bacterial reduction between the two pathogens [60,61].

### 3.5. pH

**Figure 2:**
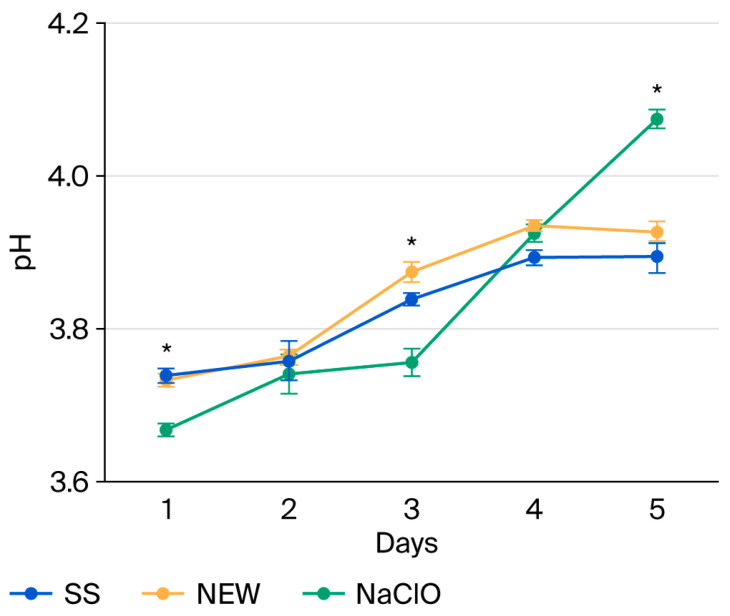
pH of treated strawberries during storage. Data points reflect the mean of five measurements ± standard deviation. * indicates significant differences (*p* ≤ 0.0001).

Strawberries are characterized by an acidic pH of 3.2 to 4.2. Under these conditions, the growth of pathogenic bacteria decreases, except for *Salmonella* spp. and *E. coli* O157:H7, which survive under these conditions [62] and they can change pH by the expression of enzymes like lysine decarboxylase, present in *Salmonella*, or arginine decarboxylase in *E. coli* [21]. Figure 2 shows that pH increases over time; this could be caused by the decrease in the citric acid concentration during storage at 4 °C. When the NEW treatment was applied, the pH value was higher than that of the SS treatment on days 2, 3, and 4. Strawberries treated with NaClO showed a significantly lower pH than strawberries treated with NEW and SS on days 1, 2, 3, and 4; by day 5, the NaClO treatment caused an increase in pH, and strawberries treated with NEW showed a low pH value similar to those treated with SS.

### 3.6. Titratable Acidity

**Figure 3:**
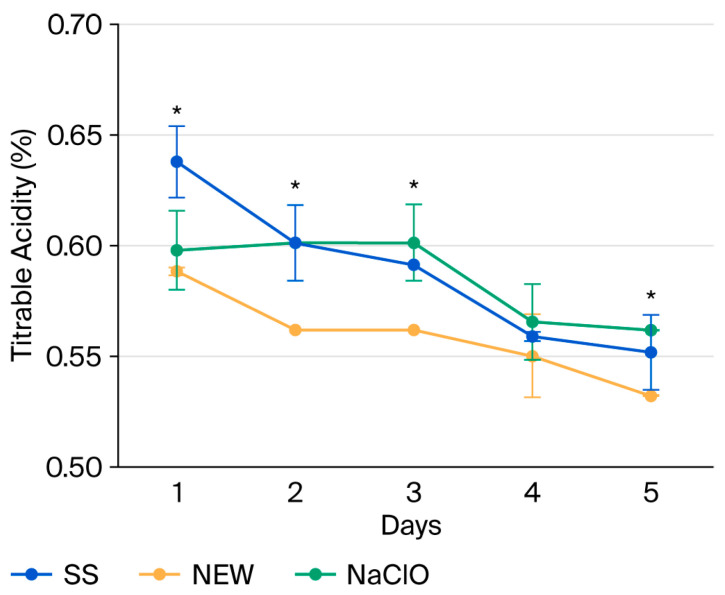
Titratable acidity of treated strawberries during storage. Data points reflect the mean of triplicate measurements ± standard deviation. * indicates significant differences (*p* ˂ 0.05).

During ripening, organic acids are transformed into sugars, with citric acid being the main organic acid in strawberries. Its content decreased over time, and therefore, the pH increased (Figure 3) [63]. It has been reported that titratable acidity should be between 0.47 and 1.28% [64]. None of the disinfection methods affects the titratable acidity values.

### 3.7. Reducing Sugars

**Figure 4:**
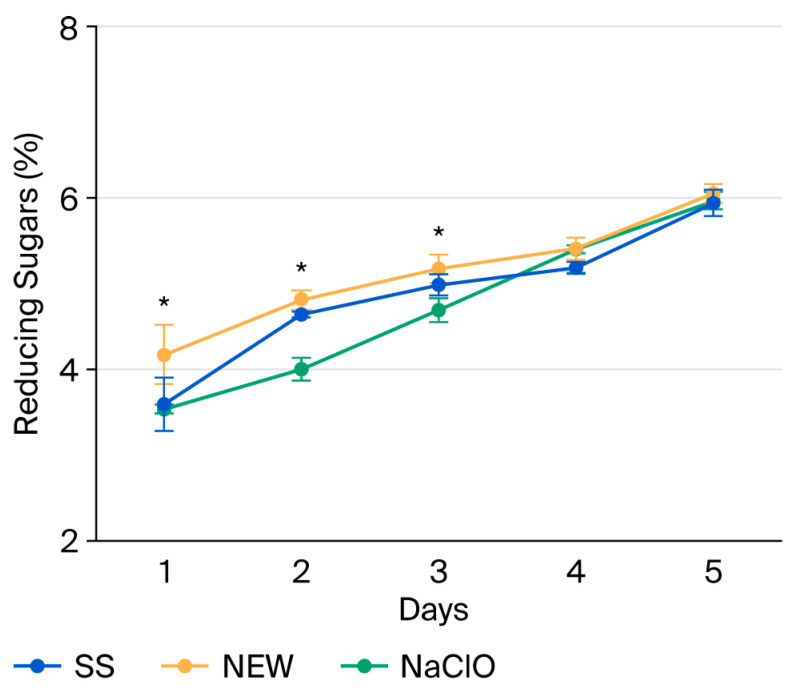
Reducing sugar content in strawberries after treatment. Data points reflect the mean of five measurements ± standard deviation. * indicates significant differences (*p* ˂ 0.0001).

The main reducing sugars are glucose and fructose, which play an important role in the freshness and sweetness of the fruit. Thus, their quantification is important, since research has shown that dark red strawberries have higher concentrations of glucose and fructose, and the sugar content is related to the quality attributes of strawberries [65]. Reducing sugars were found to increase with storage time (Figure 4); this is consistent with what was reported by del Olmo [65]. On day 0, strawberries had a completely cherry-red color and changed to a dark red during storage, which is consistent with the increase in reducing sugars.

### 3.8. Soluble Solids

Soluble solids are composed primarily of sugars, where glucose and fructose are the main components of reducing sugars. Sugar content was determined as °Bx. During fruit ripening, soluble solids increase due to the use of organic acids in gluconeogenesis [66], resulting in a decrease in citric acid and an increase in glucose and fructose. Postharvest sugar content and composition determine the shelf life, freshness, and sweetness of strawberries [65].

**Figure 5:**
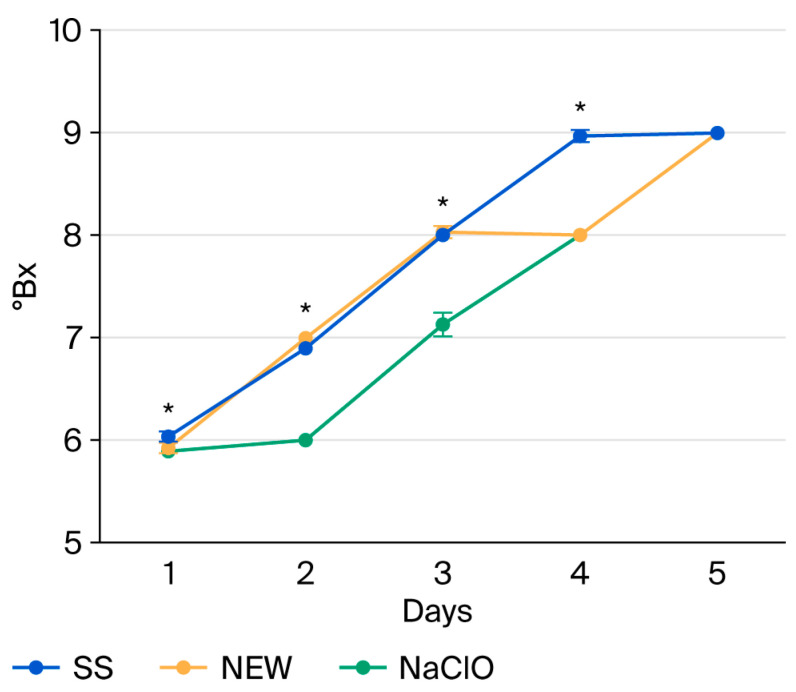
Sugar content in strawberries after treatment, with results reported as °B. Data points reflect the mean of triplicate measurements ± standard deviation. * indicates significant differences (*p* ˂ 0.0001).

After four days of storage, the NEW and NaClO solutions decreased soluble solid content in treated strawberries (Figure 5). However, at the end of the monitoring stage, all treatments reached the same °Bx value.

### 3.9. Total Phenolic Compounds

The chlorine solution and NEW showed no significant differences on days 1, 2, 3, and 5, showing the same effect on phenol content. However, a significant difference was observed between treatments on days 1, 2, and 4 compared with SS. Initially, phenolic content remained stable; therefore, on day 4, values were higher than in the SS treatment, and on day 5, no differences were observed between treatments.

**Figure 6:**
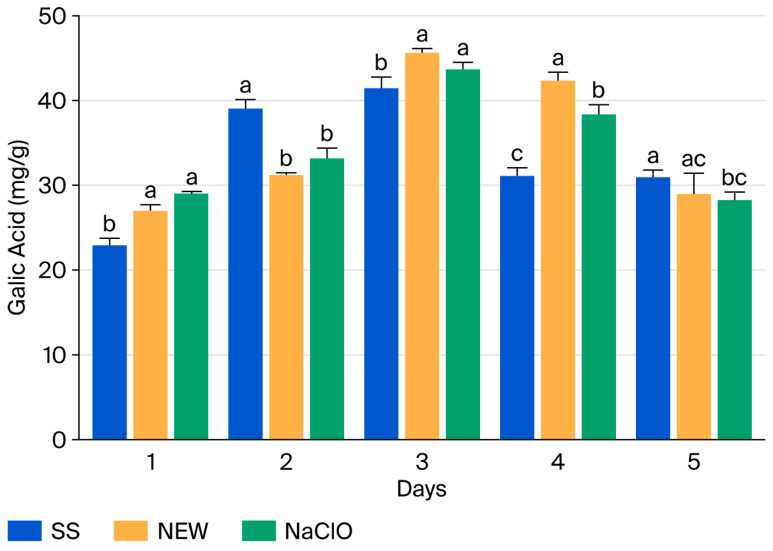
Total phenolic compounds in strawberries after treatment, with results reported as °B. Data points reflect the mean of triplicate measurements ± standard deviation. Different letters indicate significant differences (*p* ˂ 0.05).

Although several variables can modify the phenolic content, the results show that strawberries sprayed with chlorine and NEW showed a greater decrease in phenolic content on day 2 (Figure 6); this effect could be due to the high oxidation–reduction potential of these solutions.

The phenol content of strawberries was expected to gradually decrease over time due to the effect of chlorine and NEW, since phenolic compounds are very susceptible to oxidation. However, studies have shown that the ripening of fruit or other plant tissues involves a series of reactions that cause changes in the phytochemical composition of plants. Phenolic content has been reported to change during ripening: a steady decrease [67,68] or an increase at the end of ripening. The phenol content in fruit is also affected by the degree of ripeness at harvest, genetic differences, preharvest environmental conditions, and postharvest storage and processing conditions. However, its concentration varies from one plant to another and even in different organs of the same plant at different ripening stages [69].

### 3.10. Vitamin C

**Figure 7:**
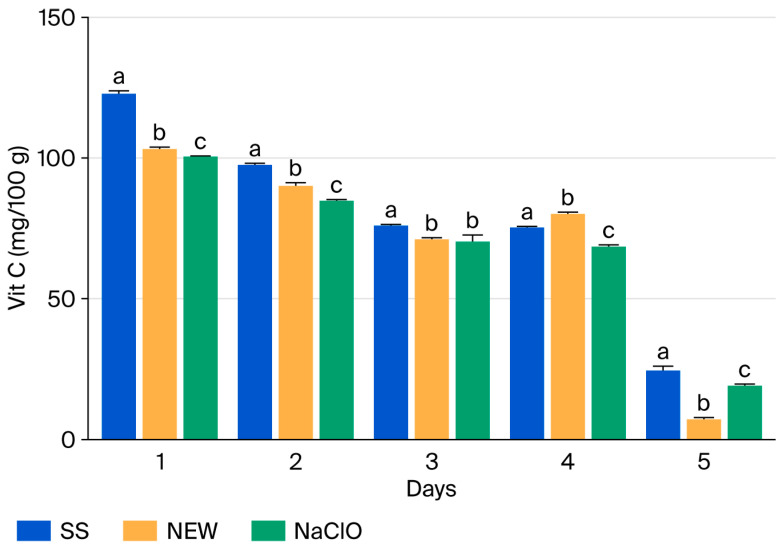
Vitamin C content in strawberries after treatment. Data points reflect the mean of triplicate measurements ± standard deviation. Different letters indicate significant differences (*p* ≤ 0.0001).

The vitamin C concentration in strawberries assessed on day 1 ranged from a maximum value of 124 mg vitamin C/100 g (water treatment) to a minimum of 7 mg vitamin C/100 g (day 5, NEW treatment) (Figure 7). The vitamin C values on day 1 are outside the range reported by Sapei [70], who indicated an approximate content of 40–70 mg vitamin C/100 g in strawberries; however, the following values of strawberry cultivars demonstrate a strong effect of genotype on vitamin C levels, with content ranging from 23 to 185 mg/100 g in different cultivars.

Despite the high vitamin C content reported on the first day of evaluation, a rapid decrease was observed in all treatments, with a similar trend. This decline is due to the fact that ascorbic acid in fruits is easily oxidized and decreases during storage. Factors influencing this oxidation process include light exposure, pH, dissolved oxygen levels, the presence of metal ions, the presence of sugar, and storage temperature [70]. Despite this, no significant differences were observed with the treatments with strong oxidizing agents (chlorine and NEW) compared to the control treatment (SS). However, the NEW-treated group had the lowest amount of vitamin C on day 5 (7.14 ± 0.45 mg vitamin C/100 g of sample), while the other treated groups had 19.20 ± 0.27 mg/ 100 g (chlorine) and 24.58 ± 1.35 mg/ 100 g (SS).

This decrease in vitamin C content when applying NEW could be attributed to the fact that the electrolyzed solution presented high oxidant activity (a property that affects ascorbic acid). A similarity in behavior was observed from day 3 to day 5 for the chlorine treatment.

Vitamin C is a sensitive vitamin because processes like preservation, storage and disinfection cause significant impacts on vitamin C losses. In one study, coriander was washed with four treatments [71] where sodium hypochlorite was included. The amount of ascorbic acid decreased by almost 50% of the content after 10 days of storage at 4 °C. In another study [72], arugula leaves (whole or chopped) were washed with acetic acid, chlorine or calcium oxide. Ascorbic acid content decreased more in chopped leaves; however, vitamin C was decreased in whole leaves, too. The highest losses were with chlorine after 5 min of treatment.

### 3.11. Color

Fruit color is important because it influences consumption decisions. It is composed of three factors. The first is luminosity, which is reported as *L*. Strawberry luminosity was determined to be unaffected by the NEW or NaClO treatments (Table 3). Furthermore, the results of the evaluation with the NEW treatment indicate a decrease in luminosity from day 2 to day 5 compared to the other three groups, indicating that the strawberries sprayed with NEW presented a darker color as the days passed; however, no significant difference was detected between treatments. The decrease in luminosity was due to the oxidation of phenolic compounds, such as anthocyanins, which are natural pigments responsible for the reddish color of strawberries and are distributed throughout the fruit [73]. NEW has high oxidative activity, and it has been reported that EW can inactivate the effect of polyphenol oxidase (PPO) and peroxidase (POD) [74,75], which enhance the oxidation reactions that cause the browning of fruits and vegetables. Their activity is high in fruits with high levels of polyphenolic compounds. In the presence of oxygen, it catalyzes the oxidation of natural phenolic compounds to their corresponding quinones, which spontaneously evolve into different pigments that cause darkening. However, PPO and POD are primarily localized within intracellular compartments, and their direct involvement in browning reactions is typically enhanced when tissue disruption occurs, such as during cutting or mechanical damage [76]. Since the strawberries evaluated in this study were not cut, direct interaction between NEW and intracellular PPO or POD was likely limited. Therefore, the slight changes observed in luminosity are more plausibly attributed to surface-level oxidative reactions affecting phenolic compounds rather than direct enzymatic inactivation. This interpretation is consistent with previous reports indicating that the impact of electrolyzed water on enzymatic browning depends on tissue integrity, exposure conditions, and enzyme accessibility [13].

Parameter a* reflects the change from green (−) to red (+). The a* value decreases during storage because ripe strawberries present a decrease in anthocyanin synthesis or an increase in their degradation [77]. The intensity of the red color is affected by changes in the synthesis, degradation, or modifications in the type of anthocyanins due to changes in external factors that affect their metabolism. After harvest, strawberries do not show an increase in respiration or ethylene production because they are non-climacteric fruits; however, there are changes in color or softening even postharvest.

Parameter a* was not different on days 1 to 4. Nevertheless, the a* value on day five was the lowest when strawberries were treated with NEW. Anthocyanins are antioxidant molecules responsible for the red pigment in strawberries; 80% of the anthocyanins present in strawberries are pelargonidin 3-glucoside and 12% are cyanidin 3-glucoside [64]. Their stability depends on pH, temperature, the presence of oxygen, and ascorbic acid content; they are stable at acidic pH values, and at alkaline pH values, the red flavyl cation is transformed into a colorless chalcone. However, strawberries treated with NEW showed a low a* value, and the SS-treated strawberries showed the highest values. This effect could be caused by the ORP activity of the evaluated solutions, which could be responsible for anthocyanin oxidation [78]. The ORP values were 878.65 and 380 for NEW and SS, respectively. These results show that ORP has a higher impact than pH on strawberry color.

Color parameter b* is used as an indicator of the change in color from blue (−) to yellow (+). In unripe strawberries, a white-yellow hue is observed due to the low concentration of anthocyanins in the fruit. As a result of the ripening process, the synthesis of these compounds increases, and therefore, during storage, the fruit turns red, decreasing the yellowish coloration, so the b* value decreases [63].

After all treatments, the ΔE values were 8.19, 6.28, and 10.41 for the SS, NaClO, and NEW treatments, respectively. Strawberries treated with electrolyzed water showed a greater color change than those treated with NaClO and SS. EW is an oxidizing solution that promotes dark coloration, resulting in a total color change of 10.41%. The total color change for NaClO was lower than that shown with electrolyzed water. These results are consistent with those of another study [14] in which the total color change was lower in strawberries treated with NaClO at a concentration of 50 ppm and stored at 4 °C.

These results could also be due to the inactivation of the PPO enzyme with NaClO. Polyphenol oxidase causes enzymatic browning in fruit upon contact with oxygen. Other studies have shown that enzyme inactivation occurs after subjecting the fruit (blueberries and mango, respectively) to the NaClO treatment [79,80], which is consistent with the results observed in the present study.

### 3.12. Sensory Analysis

**Figure 8:**
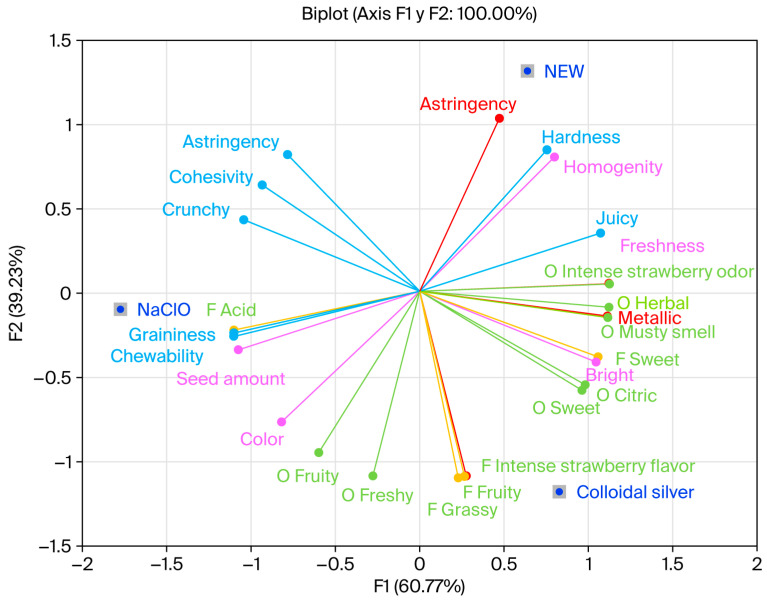
Principal component analysis results via General Procrustes Analysis of the modified flash Profile map, showing the relative sensory positioning of the appearance (pink), smell (green), taste (orange), aftertaste (red), and texture (blue) of the evaluated strawberries.

The principal component analysis (PCA) results obtained via General Procrustes Analysis (GPA) show that the F1 and F2 components explain 56.74% and 43.26% of the variability in the data, respectively (Figure 8).

Strawberries treated with NEW showed a positive and negative correlation with the F1 and F2 components, respectively; they were characterized by a fresh and homogeneous appearance. Among their attributes, the intensity of the strawberry aroma stands out, a desirable characteristic for consumers. Regarding flavor, herbaceous, sweet, intense strawberry, and fruity notes were perceived. Likewise, no correlation was observed in the aftertaste with this treatment, and a crunchy texture was perceived. These sensory attributes are commonly associated with positive consumer acceptance of fresh strawberries, as key sensory qualities such as aroma, sweetness, and texture drive liking and purchase intent in strawberry sensory evaluations [81].

Moreover, strawberries treated with ionized silver showed a negative correlation with both components. These strawberries are characterized by a shiny appearance and a fresh, herbaceous aroma. However, a metallic aftertaste was also perceived, which may negatively affect consumer preference despite acceptable appearance and aroma attributes. Strawberries treated with saline solution (SS) were not included in the sensory evaluation because saline solutions can introduce a direct salty taste that is not representative of sanitizing treatments intended for fresh produce; additionally, as untreated strawberries do not meet required food safety standards, their inclusion would not reflect commercially relevant sensory outcomes.

The disinfection treatments differed in appearance, odor, taste, aftertaste, and texture, as each disinfectant solution was located in a different quadrant of the sensory space. Overall, the sensory differences observed among treatments indicate that sanitization strategies can influence attributes directly related to consumer acceptance, such as aroma intensity, flavor quality, and texture [82].

In this context, NEW emerged as a promising alternative by combining antimicrobial efficacy with minimal negative impact on sensory properties, which is critical for consumer satisfaction and marketability of fresh strawberries. Consumer perception studies emphasize that sensory quality (aroma, taste, and texture) is a primary driver of acceptance in fresh fruits, including strawberries [83].

## 4. Conclusions

This work investigated the impact of NEW (as a disinfectant) on the physicochemical properties of strawberries during a 5-day storage period. The results demonstrated its influence on different strawberry properties and attributes.

NEW and sodium hypochlorite proved to be solutions with effective bactericidal activity on strawberries contaminated with *Salmonella* Typhimurium and *Escherichia coli* O157:H7, when both solutions were applied by spraying. These findings support the potential of NEW as an alternative sanitizing treatment for maintaining food safety in fresh produce.

This study evaluated the impact of NEW on the physicochemical properties of strawberries, compared with NaClO and saline solution. The pH increased more markedly in strawberries treated with NaClO, whereas NEW did not induce significant changes in pH. Titratable acidity and vitamin C content decreased during storage in all treatments, with a measurable reduction in vitamin C observed after NEW application, indicating that although changes were within ranges commonly reported during postharvest storage, NEW may influence certain labile nutritional components.

The reducing sugar content of strawberries was less affected by the chlorine solution compared to that of strawberries treated with NEW.

Phenolic compound content initially increased and then decreased after day 4; strawberries treated with NaClO and NEW exhibited a more pronounced decline by day 5. Despite these variations, the antioxidant capacity of strawberries was less affected by NEW compared to NaClO, suggesting a milder impact on bioactive compounds.

The values of a* decreased slightly in strawberries treated with NEW, although no statistically significant differences were detected among treatments. Overall, these results indicate that NEW induces minor but measurable changes in specific physicochemical parameters, such as vitamin C content and color attributes, without causing severe deterioration of strawberry quality

The application of disinfectant solutions (NaClO, ionized silver, and NEW) resulted in changes in the sensory profile of strawberries; NEW was characterized by desirable attributes, including a fresh and uniform appearance and an intense strawberry aroma and flavor.

Considering its antimicrobial efficacy and its limited impact on physicochemical and sensory quality, NEW represents a promising alternative for the disinfection of ready-to-eat strawberries.

Nevertheless, further studies are required to optimize application conditions, evaluate long-term storage effects, and assess consumer acceptance under commercial conditions.

## Figures and Tables

**Table 1:** Physicochemical characteristics of solutions.

	SS	NEW	NaClO
pH	6.90 ± 0.06	6.24 ± 0.03	7.45 ± 0.01
ORP (mV)	380 ± 1.43	878.65 ± 0.88	820.11 ± 1.74
Cl_2_ (ppm)	ND	54.96 ± 0.58	141.80 ± 0.06

Values represent the mean ± SD (n = 3). ND, not detectable.

**Table 2:** Bacterial viable count (log CFU/mL).

	SS	NEW	NaClO
*Salmonella* Typhimurium	9.39 ± 0.10	N.D.	5.16 ± 0.28
*Escherichia coli* O157:H7	9.30 ± 0.06	N.D.	N.D.

N.D., not detected. Detection limit: log CFU/mL = 3. Values represent the means ± SD.

**Table 3:** Color parameters of strawberries after storage period.

	L*			a*			b*		
Day	NEW	NaClO	SS	NEW	NaClO	SS	NEW	NaClO	SS
1	35.89 ^a^	34.57 ^a^	36.15 ^a^	32.55 ^ab^	30.83 ^b^	33.35 ^a^	18.71 ^a^	17.92 ^a^	19.98 ^a^
2	34.79 ^a^	35.98 ^a^	35.75 ^a^	33.33 ^a^	32.92 ^a^	32.64 ^a^	18.47 ^a^	10.08 ^a^	17.64 ^a^
3	35.33 ^a^	36.23 ^a^	35.15 ^a^	29.98 ^b^	32.28 ^a^	31.87 ^ab^	15.11 ^b^	19.68 ^a^	16.04 ^b^
4	33.65 ^a^	34.45 ^a^	34.55 ^a^	28.67 ^a^	29.91 ^a^	29.99 ^a^	14.05 ^a^	14.07 ^a^	15.58 ^a^
5	32.51 ^a^	32.71 ^a^	33.71 ^a^	25.57 ^c^	23.73 ^b^	29.76 ^a^	11.76 ^a^	12.78 ^a^	13.03 ^a^

Values represent the means ± SD within a row without a common superscript are statistically different (*p* < 0.05).

## Data Availability

The original contributions presented in this study are included in the article and Supplementary Materials. Further inquiries can be directed to the corresponding author.

## Supplementary Materials

The following supporting information can be downloaded at: https://www.mdpi.com/article/10.3390/foods15050800/s1. Bactericidal statistical analysis and standard curve for glucose quantification are included in supplemental materials.
