# Supplementary material for: Neutral Electrolyzed Water as Sanitizer Solution in Fresh Foods: The Strawberry as a Study Model
Source: Foods. 2026 Feb 24;15(5):800. doi: 10.3390/foods15050800 (PMC12985101; doi:10.3390/foods15050800)
Supplement: Supplementary file 1 [file foods-15-00800-s001.zip › foods-4134182-supplementary.pdf]

Supporting Information

Tukey's multiple comparisons test	Mean Diff.	95% CI of diff.	Significant?	Summary	Adjusted P Value
SS vs. NEW	1.487	0.9750 to 1.998	Yes	**	0.0061
SS vs. NaClO	1.287	0.0008552 to 2.572	Yes	*	0.0499
NEW vs. NaClO	-0.2000	-1.136 to 0.7357	No	ns	0.5345

Figure S1. Statistic analysis of treatments in contaminated strawberries with *Salmonella* Typhymurium.

Tukey's multiple comparisons test	Mean Diff.	95% CI of diff.	Significant?	Summary	Adjusted P Value
SS vs. NEW	2.117	1.861 to 2.372	Yes	****	< 0.0001
SS vs. NaClO	1.307	0.6774 to 1.936	Yes	*	0.0121
NEW vs. NaClO	-0.8100	-1.372 to -0.2481	Yes	*	0.0247

Figure S2. Statistic analysis of treatments in contaminated strawberries with *Escherichia coli*.

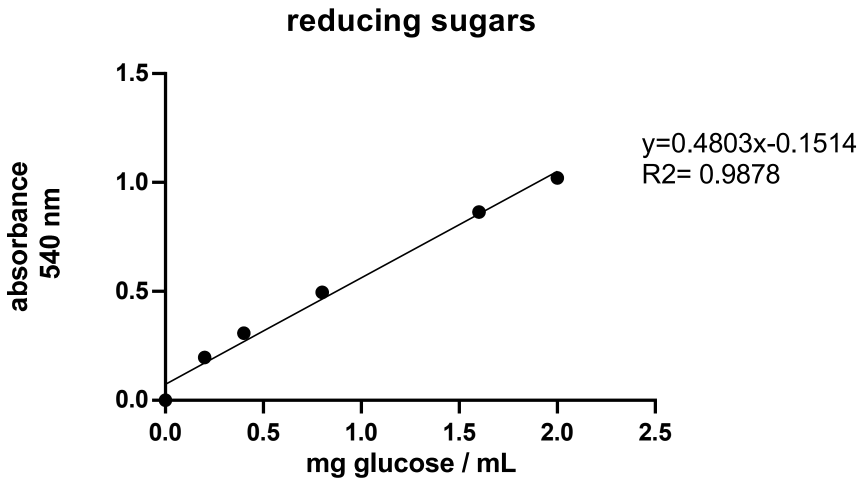

Figure S3. Glucose standard curve.
